# Volumetric Analysis of Hearing-Related Structures of Brain in Children with GJB2-Related Congenital Deafness

**DOI:** 10.3390/children9060800

**Published:** 2022-05-30

**Authors:** Matthias W. Wagner, Sharon L. Cushing, Makabongwe Tshuma, Karen A. Gordon, Birgit B. Ertl-Wagner, Logi Vidarsson

**Affiliations:** 1 Department of Diagnostic Imaging, Division of Neuroradiology, The Hospital for Sick Children, Toronto, ON M5G 1X8, Canada; makabongwe.tshuma@cw.bc.ca (M.T.); birgitbetina.ertl-wagner@sickkids.ca (B.B.E.-W.); logi.vidarsson@sickkids.ca (L.V.); 2 Department of Medical Imaging, University of Toronto, Toronto, ON M5S 1A1, Canada; 3 Department of Otolaryngology, Head & Neck Surgery, The Hospital for Sick Children, Toronto, ON M5G 1X8, Canada; sharon.cushing@sickkids.ca (S.L.C.); karen-a.gordon@sickkids.ca (K.A.G.); 4 Department of Otolaryngology, Head & Neck Surgery, University of Toronto, Toronto, ON M5S 1A1, Canada; 5 Archie’s Cochlear Implant Laboratory, The Hospital for Sick Children, Toronto, ON M5G 1X8, Canada; 6 Imaging Department, British Columbia Children’s Hospital, Vancouver, BC V6H 3N1, Canada; 7 Department of Communications Disorders, The Hospital for Sick Children, Toronto, ON M5G 1X8, Canada

**Keywords:** hearing loss, GJB2, FreeSurfer, Heschl’s gyrus, children

## Abstract

Background: Children with non-syndromic hereditary sensorineural hearing loss (SNHL) provide an opportunity to explore the impact of hearing on brain development. Objective: This study investigates volumetric differences of key hearing-related structures in children with gap junction protein beta 2 GJB2-related SNHL compared to controls. Materials and methods: Ninety-four children with SNHL (*n* = 15) or normal hearing (*n* = 79) were studied using automated volumetric segmentation. Heschl’s gyrus (HG), anterior HG (aHG), planum temporale (PT), medial geniculate nucleus (MGN), and nucleus accumbens (NA) were analyzed relative to total brain volume (TBV) at two different age groups: (1) 7–12 months and (2) 13 months–18 years. Two-sided t-tests were used to evaluate differences between groups. Differences were considered significant if *p* < 0.007. Results: Significantly smaller aHG-to-TBV ratios were found in 13-month-to-18-year-old patients (*p* < 0.0055). HG-, PT-, MGN-, and NA-to-TBV ratios were smaller in the same age group, without reaching a significant level. Conversely, HG- and NA-to-TBV were larger in the younger age group. No significant differences were found between the groups for age and TBV. Conclusions: In this exploratory volumetric analysis of key hearing-related structures, we observed age-related changes in volume in children with GJB2-related SNHL.

## 1. Introduction

Hearing loss (HL) affects approximately 2–3 infants in 1000 live births [1]. Broadly, hearing loss can be classified according to (1) type, including conductive, sensorineural, and mixed HL; (2) onset, including pre-lingual or post-lingual; (3) severity; or (4) acquired vs. hereditary causes [2]. Hereditary HL is further classified into syndromic and non-syndromic etiologies. For non-syndromic hereditary HL, the most common mutation occurs in the gap junction protein beta 2 gene (GJB2), which accounts for up to 50% of autosomal recessive HL and 20% of all hereditary HL [3]. The GJB2 gene encodes for a gap junction protein (connexin 26), which is crucial for the regulation of the passage of potassium ions and inner ear homeostasis [3,4].

In the adult population, several studies assessed the relationship between SNHL and gray matter (GM) and brain volume in the primary auditory cortex (PAC) region [5,6,7,8], which is included in the Heschl’s gyrus (HG) [9]. SNHL was associated with decreased GM volume in the PAC [5,6] and decreased total brain and white matter (WM) volumes [8]. Recently, Jiang et al. assessed WM in children with congenital SNHL using tract-based spatial statistics analysis [10]. They found higher axial and radial diffusivity in the corticospinal tract and corpus callosum, and the anterior thalamic radiation in children with SNHL compared to controls. Additionally, fractional anisotropy was lower in the HG in children with SNHL, and there was a negative correlation between mean fractional anisotropy values in the HG and age [10]. These findings are suggestive of decreased microstructural integrity and/or abnormal white matter maturation of the acoustic radiation in children with SNHL [10]. Another study with congenitally deaf adults showed decreased WM volume in the HG compared to controls [11], suggesting that a congenital lack of auditory stimuli could result in decreased myelination and fiber density connected to the auditory cortices [10,11]. Several publications assessed WM or GM volume in children with non-syndromic HL [10,12,13,14,15,16,17]. Two of these studies evaluated the volume of the HG [12,14], and one study assessed the nucleus accumbens (NA), which is an auditory gain controller important in the behavioral and emotional response to environmental sounds [14]. To our knowledge, the volume of other key structures in the central auditory system, including the medial geniculate nucleus (MGN), which serves as a relay station between the PAC and the inferior colliculus [18], and the planum temporale (PT), which is considered the secondary auditory cortex [19], have not been studied in children with non-syndromic SNHL. We, therefore, aimed to assess the volume of key structures of hearing, including the HG, MGN, PT, and NA in children with GJB2-related congenital SNHL, and compare them to age- and gender-matched controls.

## 2. Material and Methods

### 2.1. Study Population

This retrospective study was approved by the research ethics board of The Hospital for Sick Children (REB 1000007199). Due to the retrospective nature of the study, informed consent was waived by the local research ethics board. All patients were identified from the electronic health record database from January 2010 to December 2019. Patient inclusion criteria were the following: (1) 0–18 years of age, (2) positive GJB2 status with severe or profound SNHL as defined by Clark et al. [20], and (3) availability of a brain MRI with a non-motion-degraded volumetric T1-weighted sequence as part of their assessment for cochlear implant candidacy due to inadequate rehabilitation with hearing aids. Demographic data of the patients were collected by a review of their clinical histories. Age- and gender-matched controls were selected from the local MRI database at SickKids using the following criteria: (1) normal MR imaging examination of the brain, (2) absence of hearing loss, (3) absence of neurological disorders, and (4) availability of a non-motion-degraded volumetric T1-weighted sequence.

### 2.2. MRI Acquisition

The entire study population underwent brain MR imaging at 1.5T or 3T across various scanners (Signa HDxt, GE Healthcare, Waukesha, WI, USA; Achieva, Philips Healthcare, Best, The Netherlands; Magnetom Skyra, Siemens Healthineers, Erlangen, Germany) with a dedicated head-coil. For each subject, high-resolution, volumetric T1-weighted 3D gradient-echo images were acquired, either in the sagittal or the axial plane: TR/TE, 6–9/2–5 ms; flip angle: 10–15; resolution: 0.85–1.1 × 0.85–1.1 × 0.9–1.1 mm; field of view (FOV): 22–24 cm; 150–200 slices.

### 2.3. Analysis: Manual Inspection and FreeSurfer Analysis

**Figure 1:**
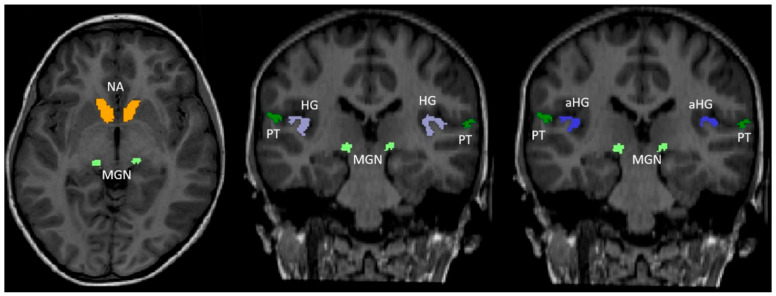
Anatomical structures used in the FreeSurfer analysis overlaid on a coronal reformat of the T1-weighted sequence. Axial and two coronal reformat of the T1-weighted sequence show the color-coded hearing-related brain structures we studied: Heschl’s gyrus (HG, purple, anterior HG (aHG, blue), planum temporale (PT, dark green), medial geniculate nucleus (MGN, light green), and nucleus accumbens (NA, orange).

FreeSurfer is a widely used, open-access software package for structural brain imaging analysis [21,22,23]. It serves as an automatic reconstruction pipeline for the processing of brain images, including skull stripping, motion artifact correction, B1 bias field correction, gray–white matter segmentation, and region labeling on the cortical surface using different atlases in pediatric [24,25] and adult age groups [26,27,28]. Technical details are available from prior publications [29,30,31]. De-identified T1 structural examinations were made available from our local picture archiving and communication system (PACS) and processed using FreeSurfer version 7.1.1 (Massachusetts General Hospital, Harvard Medical School; http://surfer.nmr.mgh.harvard.edu (accessed on 1 September 2020)) [29], which aligns the input examination to the Desikan–Killiany [26], Destrieux [27], and Iglesias [32] atlases. Experience has shown that FreeSurfer has a high failure rate with data from patients younger than 1 year old. Zöllei et al. developed Infant FreeSurfer [24] to address this problem, incorporating age-matched templates to produce reliable automated brain segmentation in infants. However, infant FreeSurfer aligns the input examinations to the Desikan–Killiany [26] atlas only. To manage the processing, we wrote our in-house, web-based, data management software running on the FreeSurfer server (Lenovo Legion C730 with i9 processor and 32 Gb of RAM (Lenovo, Quarry Bay, Hong Kong)). The anatomical regions of interest (ROI) included the available regions of the brain involved in the auditory system, including the anterior HG (aHG), HG, PT, MGN, and NA. The aHG and PT were extracted from the Destrieux atlas (children aged 13 months to 18 years), while the HG and NA came from the Desikan–Killiany atlas (all study participants). The Iglesias atlas was used to extract the MGN (children aged 13 months to 18 years). Whole-brain volume was obtained from the Desikan–Killiany atlas for all study participants. The inferior colliculi are part of the central auditory system but were not assessed, since they are not included in the FreeSurfer atlases as a separate anatomical region. The parcellated output was displayed with the respective label map overlay and images were visually inspected for quality of regional segmentation results (Figure 1).

### 2.4. Statistical Analysis

Statistical analysis was conducted using Matlab (Mathworks, Nattick, MA, USA). Patients and age- and gender-matched controls were divided into two groups based on the availability of FreeSurfer-derived ROIs: (1) 7–12 months of age and (2) 13 months to 18 years. In order to standardize age-related volumetric differences, ROI volumes were divided by the total brain volume (TBV) for all subjects. Further, ROI volumes of the left and right hemispheres were pooled and, given the limited number of GJB2, confirmed profound hearing loss cases with suitable imaging. Two-sided t-tests were used to assess differences in age for patients and controls. For each age group, the FreeSurfer-derived ROI volumes divided by the TBV were compared. A *p*-value, calculated using Student’s *t*-test, was used to assess statistical significance. All tests were two-sided, and observed differences were considered statistically significant if *p* ≤ 0.007. This value corresponds to a Bonferroni-corrected *p*-value threshold of 0.05 [33]. 

## 3. Results

### 3.1. Study Population

We identified 19 children with GJB2-related congenital HL. One child was excluded because of an unavailable pre-contrast volumetric T1-weighted sequence. Three children were excluded because FreeSurfer failed to provide accurate segmentations on manual inspection. Therefore, the final patient cohort consisted of 15 patients.

There were 4 children in the 7–12 month group (mean age: 8 months, 2 females) and 11 children in the 13-month-to-18-year group (mean age: 9 months, 4 females). All affected patients had bilateral HL. Thirteen patients had profound, and two had severe hearing loss. The control cohort consisted of 79 children (mean age: 74 months, 31 females): 9 children in the 7–12-month group (mean age: 9 months, 4 females) and 70 in the 13-month-to-18-year group (mean age: 74 months, 31 females). The mean age of the entire cohort of 94 patients and controls was 65 months (42 females). No significant differences were found between patients and controls in each of the groups for age (*p* = 0.12). Demographic information for patients and controls is summarized in Table 1. The children included had hearing loss bilaterally. Pure tone averages of hearing thresholds at 500, 1000, and 2000 Hz were mean (SD) = 93.6 (13.1) dB HL in the left ear and 92.5 (21.3) dB HL in the right ear. All received cochlear implants after a period of hearing aid use. Eleven children received bilateral cochlear implants (nine simultaneously; two sequentially), and four used a cochlear implant in the left ear and a hearing aid in the right ear. Age at first implant was mean (SD) = 5.0 (4.7) years. Data logs available from the cochlear implants show consistent use of mean (SD) = 11.5 (2.0) hours daily.

### 3.2. Volumetric Results

TBV was not significantly different for patients compared to controls in both age groups: 7–12 months: 0.64 L vs. 0.69 L, *p* = 0.29, 13 months to 18 years: 1.1 L vs. 1 L, *p* = 0.25. Volumetric measurements for children with HL and age- and gender-matched controls are summarized and compared in Table 2. Individual volumes are shown in Supplementary Table S1.

Heschl’s gyrus: In the 7–12-month age group, mean volumes relative to the intracranial volume for patients and controls were 0.9 parts per million (ppm) and 0.75 ppm. In the 13-month-to-18-year age group, mean volumes relative to the intracranial volume for patients and controls were 0.9 ppm and 1.0 ppm.

Anterior Heschl’s gyrus: The aHG was assessed in the older patient group only. In the 13-month-to-18-year age group, mean volumes relative to intracranial volumes for patients and controls were 0.8 ppm and 1.0 ppm (*p* = 0.0055).

Planum temporale: The PT was assessed in the older cohort only (13 months to 18 years). Mean volumes relative to intracranial volumes for patients and controls were 1.4 ppm and 1.5 ppm (*p* = 0.2).

Medial geniculate nucleus: The MGN was assessed in the older age group only (13 months to 18 years). Mean volumes relative to intracranial volume for patients and controls were 0.08 ppm and 0.09 ppm (*p* = 0.11).

Nucleus accumbens: The NA was assessed in both age groups. In the 7–12-month age group, mean volumes relative to intracranial volume for patients and controls were 0.3 ppm and 0.28 ppm (*p* = 0.2). In the 13-month–18-year age group, mean volumes relative to intracranial volume for patients and controls were 0.45 ppm and 0.5 ppm (*p* = 0.2).

## 4. Discussion

In this exploratory volumetric analysis of key hearing-related structures, we observed age-related changes in volume in children with GJB2-related SNHL. We found significantly smaller aHG/TBV ratios in 13-month-to-18-year-old patients with GJB2-related SNHL, compared to controls, in agreement with Tae et al. [14]. This relation was reversed in the 7–12 month age group, with an increased HG/TBV ratio in patients exceeding the control cohort, as previously shown by Smith et al. [12]. Further, we found larger HG-, PT-, MGN-, and NA-to-TBV ratios in patients aged 13 months to 18 years, while the NA-to-TBV ratio was smaller in the younger age group. These findings further support the suggestion of an initial synaptic immaturity with an increased GM thickness in infants [12], and subsequent underdevelopment of hearing-related structures in older children [34].

In normal development, the GM density declines with age and is larger for younger individuals [12,35]. Smith et al. showed that there is a greater GM volume at the anterior HG in children with hearing impairment [12]. Their cohort of 16 children with hearing impairment had a mean age of 14 months. The findings in our group of 7–12 months old children (mean age: 8 months) are in agreement with this observation. Additionally, we showed that the volume of the aHG is lower in children with congenital SNHL above 13 months of age. This finding can be explained by the lack of peri- and post-natal sound stimulation, which is critical for the normal synaptogenesis of the PAC [36]. In developing children, the PAC reaches its peak synaptic density approximately 3 months after birth [37]. This is followed by synapse elimination, which continues into childhood [37,38]. A similar course of delayed synaptic peak density was also shown in hearing-impaired cats [36]. Our findings further support the hypothesis that, due to the lack of sound stimulation during early infancy, there is a delayed formation of synapses in the PAC, and the subsequent trajectory of synaptic elimination (pruning) is disturbed [12]. 

A strength of our study was the very homogeneous patient cohort. GJB2-related congenital SNHL is established in an individual with mild-to-profound congenital, mostly non-progressive sensorineural hearing impairment, and identification of bi-allelic pathogenic variants in GJB2, which encode connexin 26 [39]. Connexin 26 is a major component of the gap junction system in the cochlea, with important roles in potassium recycling, intercellular calcium signaling, and electrical coupling to support electro-motile cochlear amplification [40,41,42]. Affected individuals have normal vestibular functions and do not experience balance problems. They learn to sit and walk at age-appropriate times and, with the exception of hearing impairment, they are healthy, and their life span is normal [39]. Other common etiologies of congenital SNHL include acquired forms often grouped as TORCH (toxoplasmosis, others, rubella, cytomegalovirus (CMV), herpes simplex viruses) infections. Besides a variable degree of hearing impairment, particularly CMV, rubella and toxoplasmosis can cause a number of CNS injuries, including migrational abnormalities, altered white-matter myelination, microcephaly, calcifications, parenchymal volume loss, and others. In light of the absence of comorbid neurological findings, non-syndromic congenital SNHL appears more suitable for the analysis of structural brain differences in central hearing-related structures.

There are limitations of our study that need to be considered when interpreting the data. First, our sample was relatively limited but comparable to prior volumetric studies in children with SNHL. In addition, prior studies assessed hearing-impaired individuals with largely heterogeneous etiologies and/or comorbid neurological findings [12,13,43]. Nevertheless, pooling of the ROI volumes of the left and right hemispheres may obscure potentially important effects of hemispheric differences in auditory cortical morphometry. Second, different scanners and field strengths were used for data acquisition. Both the GJB2 group and our Normal group were clinical patients; there are no systemic differences in how they were managed at our imaging clinic. Further, dividing our limited sample size into groups based on imaging parameters is unlikely to yield statistically sensible results. However, others have investigated the impact of field strength and imaging parameters on FreeSurfer. Heinen et al. [44] compared FreeSurfer volume estimates between 1.5T and 3T scanners and found robust volume estimates between field strength, with differences in GM volume of only 1%. Furthermore, Wonderlick et al. found FreeSurfer to be fairly robust in imaging parameter selection, although some bias was observed when anisotropic MRI data was used [45]. Third, FreeSurfer performs parcellation based on the Desikan–Killiany [26], Destrieux [27], and Iglesias [32] atlases, etc. However, Infant FreeSurfer only uses the Desikan–Killiany [26] atlas. Therefore, we were unable to study the volumes of aHG, PT, and MGN in the 7–12-month-old group.

## 5. Conclusions

In conclusion, in this homogeneous group of children with GJB2-related SNHL, we observed age-related differences in GM volumes of key hearing-related structures, including aHG/HG, PT, MGN, and NA, compared to normal-hearing children. As GJB2-mutations cause isolated hearing loss, this can be used as a reference to differentiate changes related to congenital hearing loss from neurodevelopmental changes in complex syndromes.

## Figures and Tables

**Table 1:** Demographics for children with GJB2-related hearing loss and controls.

	Age Group	7–12 Months	13 Months to 18 Years
Number of subjects	GJB2	4	11
	Controls	9	70
Number of males	GJB2	2	6
	Controls	5	39
Number of females	GJB2	2	5
	Controls	4	31
Average age (range) (months)	GJB2	8 (7–9)	73 (14–177)
	Controls	9 (7–12)	74 (15–187)
	*p*-value	0.12	0.47
Brain volume (L)	GJB2	0.64	1.1
	Controls	0.69	1
	*p*-value	0.29	0.25

Legend: Overview of age and gender in different age groups for patients and controls.

**Table 2:** FreeSurfer results of GJB2 patients and controls. The group-average standard deviation is reported in parenthesis.

Age Group		7–12 Months	13 Months to 18 Years
Heschl’s gyrus (%)	GJB2	0.09(0.02)	0.09(0.02)
	Controls	0.075(0.02)	0.1(0.02)
	*p*-value	0.09	0.02
Anterior Heschl’s gyrus (%)	GJB2	N/A	0.08(0.02)
	Controls	N/A	0.1(0.02)
	*p*-value	N/A	0.0055
Planum temporale (%)	GJB2	N/A	0.14(0.05)
	Controls	N/A	0.15(0.05)
	*p*-value	N/A	0.2
MGN (%)	GJB2	N/A	0.008(0.002)
	Controls	N/A	0.009(0.002)
	*p*-value	N/A	0.11
Nucleus accumbens (%)	GJB2	0.03(0.01)	0.05(0.01)
	Controls	0.03(0.01)	0.05(0.01)
	*p*-value	0.2	0.2

Legend: Results for patients and controls. Values are in percent unless otherwise noted. For the anterior Heschl’s Gyrus, the planum temporale and medial geniculate nucleus results are not available for the 7–12-month age group, as infant FreeSurfer only aligns to the Desikan–Killiany atlas.

## Data Availability

The datasets generated during and/or analyzed during the current study are available from the corresponding author on reasonable request.

## Supplementary Materials

The following supporting information can be downloaded at: https://www.mdpi.com/article/10.3390/children9060800/s1.
