# Supplementary material for: Volumetric Analysis of Hearing-Related Structures of Brain in Children with GJB2-Related Congenital Deafness
Source: Children (Basel). 2022 May 30;9(6):800. doi: 10.3390/children9060800 (PMC9222099; doi:10.3390/children9060800)
Supplement: Supplementary file 1 [file children-09-00800-s001.zip › children-1707981-supplementary.pdf]

Supplementary Table 1. Individual volumetric results of GJB2 patients (top Desikan-Killiany atlas results, bottom Destrieux atlas results).

Patient	Gender	Age (mo)	Brain Volume [L]	Heschl gyrus [%]	Anterior Heschl gyrus [%]	Planum temporale [%]	MGN [%]	Nucleus accumbens [%]
1	F	6	0.659	0.074%	N/A	N/A	N/A	0.028%
2	F	7	0.796	0.067%	N/A	N/A	N/A	0.034%
3	M	8	0.891	0.108%	N/A	N/A	N/A	0.040%
4	M	9	0.882	0.063%	N/A	N/A	N/A	0.025%
6-12 Mo. Normal average (std)		9	0.690	0.075(0.02)%	N/A	N/A	N/A	0.03(0.01)%
5	M	14	1.215	0.111%	0.110%	0.172%	0.008%	0.030%
6	F	21	1.006	0.143%	0.139%	0.205%	0.008%	0.050%
7	M	24	1.078	0.119%	0.112%	0.187%	0.009%	0.052%
8	M	29	1.212	0.114%	0.118%	0.117%	0.012%	0.052%
9	F	32	1.028	0.113%	0.093%	0.159%	0.012%	0.076%
10	F	51	1.633	0.080%	0.069%	0.086%	0.007%	0.036%
11	M	89	1.525	0.087%	0.082%	0.159%	0.009%	0.047%
12	M	112	1.534	0.106%	0.099%	0.189%	0.009%	0.043%
13	M	127	1.699	0.088%	0.080%	0.201%	0.009%	0.045%
14	F	140	1.397	0.093%	0.092%	0.112%	0.010%	0.043%
15	F	171	1.635	0.079%	0.074%	0.094%	0.008%	0.042%
12 Mo. - 18 year Normal average (std)		74	1.000	0.1(0.02)%	0.1(0.02)%	0.15(0.05)%	0.009(0.002)%	0.05(0.01)%
